# Functions and mechanisms of RNA tailing by nucleotidyl transferase proteins in plants

**DOI:** 10.3389/fpls.2024.1452347

**Published:** 2024-10-15

**Authors:** Jiwei Chen, Xiaozhen Li, Xianxin Dong, Xiaoyan Wang

**Affiliations:** ^1^ Center for Biological Science and Technology, Key Laboratory of Cell Proliferation and Regulation Biology of Ministry of Education, Advanced Institute of Natural Sciences, Faculty of Arts and Sciences, Beijing Normal University, Zhuhai, China; ^2^ Instrumentation and Service Center for Science and Technology, Beijing Normal University, Zhuhai, China

**Keywords:** nucleotidyl transferase protein (NTP), uridylation, mRNA, sRNA, RNA degradation, Arabidopsis

## Abstract

The addition of non-templated nucleotides at the 3’ terminus of RNA is a pervasive and evolutionarily conserved posttranscriptional modification in eukaryotes. Apart from canonical poly(A) polymerases (PAPs), which are responsible for catalyzing polyadenylation of messenger RNAs in the nucleus, a distinct group of non-canonical PAPs (ncPAPs), also known as nucleotidyl transferase proteins (NTPs), mediate the addition of uridine and adenosine or of more intricate combinations of nucleotides. Among these, HEN1 SUPPRESSOR 1 (HESO1) and UTP: RNA URIDYLYLTRANSFERASE (URT1) are the two most extensively studied NTPs responsible for the addition of uridine to the 3’ ends of RNAs (RNA uridylation). Recent discoveries have improved our understanding of the functions and mechanisms of uridylation mediated by HESO1 and URT1 in RNA metabolism. Furthermore, more NTPs have been identified to function in the 3’ tailing of RNA and not solely through uridylation. Accumulating evidence indicates that RNA tailing plays important roles in plant growth and development, stress responses, and disease resistance. In this review, we examined the latest developments in RNA tailing by NTPs, with a focus on RNA uridylation and metabolism in plants. We also discussed the essential aspects for future research in this field.

## 1 Introduction

The addition of non-templated nucleotides to the 3’ end of RNA, known as RNA tailing, is a widespread and highly conserved process in eukaryotes. Such modifications play crucial roles in regulating gene expression by modulating various RNA metabolic processes, such as maturation, degradation, and activity (De Almeida et al., 2018; Yu and Kim, 2020). Apart from polyadenylation of messenger RNAs (mRNAs) in the nucleus, which is catalyzed by canonical poly(A) polymerases (cPAPs), non-canonical tails, including uridines, adenosines, or more complex combinations of nucleotides, have been identified in various RNA molecules mainly due to the advent of high-throughput RNA sequencing technologies in recent years (De Almeida et al., 2018; Zigackova and Vanacova, 2018; Yu and Kim, 2020).

In plants, non-canonical tailings exhibit diverse functional outcomes according to distinct tailing patterns (**Table 1**). Among these modifications, 3’ uridylation is the most prevalent non-canonical form of tailing and has been extensively studied in Arabidopsis (de Almeida et al., 2018). Uridylation plays a crucial role in influencing the destiny of RNA through a variety of mechanisms, such as facilitating or inhibiting RNA processing, modifying RNA functionality, and altering RNA degradation. The principal role of RNA uridylation is to facilitate RNA degradation (De Almeida et al., 2018). 3’ adenylation was identified in ribosomal RNA (rRNA) maturation byproducts, precursors, and microRNAs (miRNAs). The adenylation of rRNA precursors contributes to their processing and degradation (Sikorski et al., 2015), whereas the adenylation of miRNAs seems to enhance their stability *in vitro* when cell extracts of *Populus trichocarpa* are utilized (Lu et al., 2009). 3’ cytidylation and guanylation have also been detected in miRNAs, mRNAs, precursor miRNAs (pre-miRNAs), and phytoviral RNAs, albeit at lower frequencies, but their precise functions remain unknown (Wang et al., 2015; Zuber et al., 2016; Song et al., 2019; Joly et al., 2023). All of these studies have revealed that RNA tailing is an integral step in the modulation of gene expression.

**Table 1:** The RNA substrates and functions of Arabidopsis NTPs.

RNA substrate	Enzyme	RNA tailing	Function	Refs
miRNA	HESO1 URT1	uridylation	Promote degradation; Modulate activity	(Fei et al., 2018; Ren et al., 2012; Tu et al., 2015; Wang et al., 2016, 2015; Yu et al., 2017; Zhai et al., 2013; Zhao et al., 2012)
	NTPs	adenylation/cytidylation/guanylation	Unknown	
pre-miRNA	HESO1	mono-/di-uridylation	Promote processing	(Song et al., 2019)
		oligo-uridylation	Promote degradation	
	HESO1	cytidylation	Unknown	
	NTP6			
	NTP7			
miRNA/miRNA* duplex	NTP4	uridylation	Enhance stability of miRNAs	(Kong et al., 2021)
het-siRNA	HESO1	uridylation	Promote degradation	(Wang et al., 2016, 2015; Zhao et al., 2012)
P4RNA	HESO1	uridylation	Enhance stability	(Ren et al., 2023)
mRNA	URT1 HESO1(?)	uridylation	Promote degradation Prevent deadenylation	(Scheer et al., 2021; Sement et al., 2013; Wang et al., 2022; Zuber et al., 2016)
	NTPs	cytidylation/guanylation	Unknown	
5’ RISC-cleavage fragment	HESO1 URT1	uridylation	Promote degradation	(Ren et al., 2014; Zhang et al., 2017; Zuber et al., 2018)
Pre-rRNA	TRL	adenylation	Promote degradation	(Sikorski et al., 2015)
	TUTase	uridylation	Unknown	
Viral RNA	HESO1 URT1 NTPs	uridylation	Promote degradation; Enhance stability ()?	(Joly et al., 2023)
	NTPs	adenylation/cytidylation/ guanylation/ mixed tailing	Unknown	

Nucleotidyl transferase proteins (NTPs), a subset of non-canonical PAPs (ncPAPs) belonging to the superfamily of DNA polymerase β-like nucleotidyltransferases, are responsible for the non-canonical tailing of RNAs. To date, NTP family genes have been identified in the genomes of various plants, including Arabidopsis, rice, maize, and soybean, comprising 10, 13, 24, and 16 putative members, respectively (Zhao et al., 2012; Yang et al., 2017; Lu et al., 2018; Kang et al., 2024). The article by de Almeida et al. provides a comprehensive review of the characteristics, classification, and evolutionary aspects of NTPs in Arabidopsis (de Almeida et al., 2018). Furthermore, an analysis of evolutionary trends and gene collinearity across various species has highlighted a relatively conserved evolutionary pattern for the NTP family, suggesting a significant and analogous role of NTPs in diverse plant species (Kang et al., 2024). Among these NTPs, HEN1 SUPPRESSOR1 (HESO1/NTP1) and UTP: RNA URIDYLYLTRANSFERASE (URT1/NTP3) are prominent terminal uridylyl transferases (TUTases) that catalyze the uridylation of various RNA species. HESO1 plays a major role in the uridylation of unmethylated miRNAs and 5’ fragments generated by the RNA-induced silencing complex (RISC) to trigger their degradation (Ren et al., 2012, 2014). In addition to its cooperative interaction with HESO1 on unmethylated miRNAs and 5’ fragments, URT1 uridylates a specific subset of deadenylated mRNAs to prevent excessive deadenylation (Sement et al., 2013; Wang et al., 2015; Zuber et al., 2018). TRF4/5-LIKE (TRL/NTP10) adenylates rRNA maturation byproducts and precursors, thereby facilitating their effective degradation or further processing by the RNA exosome (Sikorski et al., 2015). Research on NTPs in plants is still in its early stages, with numerous unresolved questions, including questions about uncharacterized NTP genes, the molecular mechanisms underlying NTP-mediated RNA metabolism, and the physiological roles of NTPs during plant development.

**Figure 1:**
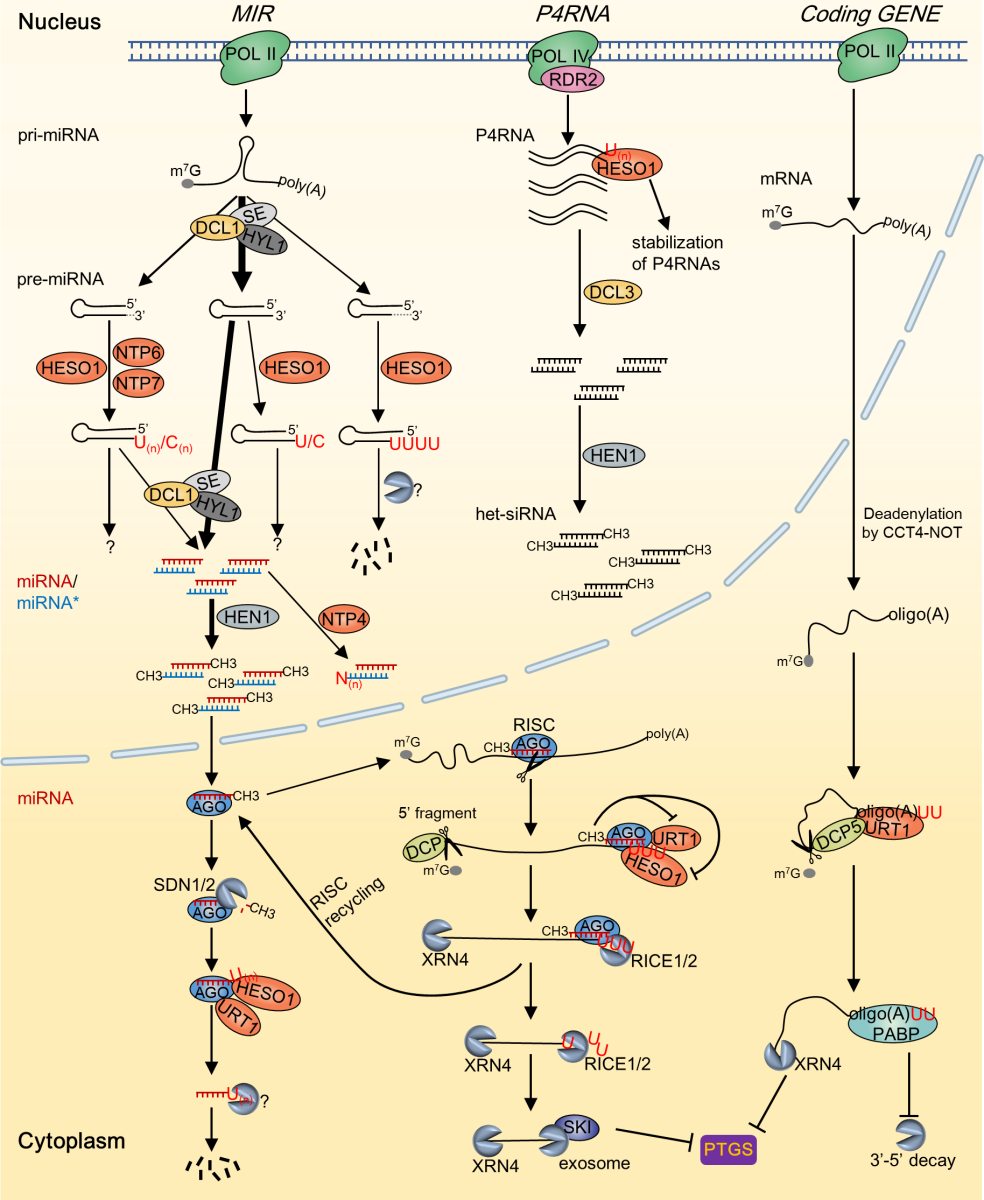
A summary of the substrates and outcomes of RNA tailing by NTPs in Arabidopsis. During the biosynthesis of sRNAs, the addition of RNA tails by various NTPs occurs in multiple stages, leading to a wide range of metabolic consequences. Deadenylated mRNA is predominantly tailed by URT1, while the 5’ RISC-cleavage fragment is primarily tailed by HESO1. These tailing processes facilitate the degradation of the substrate RNA molecules and serve to inhibit posttranscriptional gene silencing. Grey dashed lines in the 3’ end of pre-miRNA indicate the trimmed nucleotides; “N” in miRNA* of the miRNA/miRNA* duplex indicates indefinite nucleotides added by NTP4.

In recent years, an increasing variety of RNAs have been identified to undergo 3’ tailing. These include pre-miRNAs, miRNA/miRNA* duplexes, RNA polymerase IV (Pol IV)/RNA-dependent RNA polymerase 2 (RDR2)-dependent double-stranded RNAs (dsRNAs) (P4RNAs), and phytoviral RNAs (Song et al., 2019; Kong et al., 2021; Joly et al., 2023; Ren et al., 2023). In addition to identifying new RNA substrates, recent researches have revealed the involvement of more NTPs in RNA tailing. Specifically, much progress has been made in elucidating the functions and mechanisms of uridylation mediated by HESO1 and URT1 in RNA metabolism. Accumulating evidence indicates that RNA tailing, particularly uridylation, plays a crucial role in plant growth and development, stress responses, and disease resistance. Here, we provide a summary of our existing understanding of the modulation of RNA processing and stability in plants through non-canonical tailing mediated by NTPs, with a specific emphasis on RNA uridylation (**Figure 1**). Moreover, we discussed the role of NTPs during plant development and stress response, and delineated the essential aspects for prospective investigations in this field.

## 2 RNA tailing: a key player in sRNA and mRNA metabolism

### 2.1 RNA tailing modulates the metabolism of various sRNA species through multiple layers

Plant small RNAs (sRNAs) are typically 20–24-nucleotide (nt) in length and predominantly comprise miRNAs and small interfering RNAs (siRNAs) (Vaucheret and Voinnet, 2024). MiRNAs are transcribed from *MIR* genes by RNA polymerase II (Pol II). Single-stranded primary miRNAs (pri-miRNAs) are mainly processed by Dicer-like 1 (DCL1) into hairpin precursor miRNAs (pre-miRNAs), which are subsequently cleaved by DCL1 to generate miRNA/miRNA* duplexes (Ding and Zhang, 2023). SiRNAs originate from double-stranded RNA precursors that are ideally paired, and the production of heterochromatic siRNAs (het-siRNAs) and phased secondary siRNAs (phasiRNAs) are RDR protein dependent. The production of het-siRNA is initiated through Pol IV-dependent transcription. Pol IV collaborates with RDR2 to produce double-stranded P4RNAs, which subsequently undergo DCL3-mediated cleavage to generate 24-nt het-siRNAs (Huang et al., 2021). MiRNA and sRNA duplexes generated by DCLs are 2’-*O*-methylated by the methyltransferase HUA ENHANCER1 (HEN1) at the 3’ termini of both strands (Li et al., 2005). During RISC loading, only the guide strand of the duplexes is incorporated into the ARGONAUTE (AGO) protein, whereas the passenger strand is degraded.

#### 2.1.1 Uridylation of single-stranded sRNAs

Uridylation of sRNAs was initially discovered in plants, and the most well-studied function of RNA uridylation in plants is its role in triggering the degradation of sRNAs. In plants, sRNAs are typically protected by 3’ end 2’-*O*-methylation to prevent destabilization induced by uridylation. Dysfunction of HEN1 or the activity of the 3’–5’ exoribonucleases SMALL RNA DEGRADING NUCLEASES1/2 (SDN1/2), which can trim the methylated nucleotide of miRNAs, leads to the depletion of 3’ terminal methylation of sRNAs (Li et al., 2005; Ramachandran and Chen, 2008). Unmethylated sRNAs are mainly uridylated by HESO1 and subsequently degraded by currently unknown mechanisms (**Figure 1**). Similar to HESO1, URT1 interacts with AGO1 and acts as the primary TUTase responsible for uridylating miRNAs in the absence of HESO1 (Wang et al., 2015). While both HESO1 and URT1 target sRNAs, they exhibit distinct and synergistic uridylation activities on these molecules. Specifically, HESO1 plays a prominent role in uridylating miRNAs, whereas URT1 targets a limited number of miRNA substrates. This contrast elucidates why HESO1 dysfunction can rescue the phenotype of *hen1* while URT1 mutation cannot (Zhao et al., 2012; Tu et al., 2015). In addition, HESO1 preferentially targets miRNAs that terminate with uridine, while URT1 tends to target miRNAs that end with adenine. This bias is consistent with the finding that URT1 serves as the primary TUTase responsible for uridylating deadenylated mRNAs *in vivo* (Sement et al., 2013). It is likely that URT1 mainly mediates mono-uridylation of unmethylated miRNAs, generating U-terminating substrates that may undergo further uridylation by HESO1 (Tu et al., 2015). Furthermore, het-siRNAs undergo uridylation mediated by HESO1 rather than URT1 in *hen1* (Zhao et al., 2012; Wang et al., 2015). Notably, although the majority of sRNAs undergo methylation by HEN1 in plants, there are certain exceptions. For instance, miR158 and miR319a in Arabidopsis, along with miR1510 in Phaseoleae species, exhibit notable 3’ end tailing even in the presence of a competent HEN1 (Zhao et al., 2012; Zhai et al., 2013; Wang et al., 2015; Fei et al., 2018). These findings imply that certain sRNA duplexes might not be effectively recognized by HEN1 for processing. Moreover, HESO1 and URT1-independent non-U tails were also identified among the miRNAs, indicating the involvement of other NTPs in non-canonical tailings (Wang et al., 2015).

In addition to its primary function of triggering degradation, uridylation also plays additional roles in sRNA metabolism. The mono-uridylation of miR158 by URT1 results in a reduced ability to effectively suppress its target in *hen1*. Furthermore, the uridylation of AGO1-bound miR165/6 by URT1 markedly diminishes its slicing activity (Tu et al., 2015). Another function of miRNA uridylation is to initiate the biogenesis of phasiRNAs, which are triggered by the cleavage of target RNAs by certain 22-nt miRNAs. Mono-uridylation of miR171a to 22-nt by URT1 confers its ability to initiate phasiRNA production in *hen1* (Tu et al., 2015). Moreover, mono-uridylation of miR1510 in Phaseoleae species by HESO1 in the wild-type (Wt) background can trigger the biogenesis of phasiRNAs from numerous *nucleotide-binding leucine-rich repeat* (*NB-LRRs*) loci (Fei et al., 2018). These examples suggest that the uridylation of sRNAs may have unique functions that extend beyond simply hastening sRNA degradation.

#### 2.1.2 Tailing on the DCL1-processed dsRNA termini

Pre-miRNA is the key intermediate during the processing of pri-miRNA to mature miRNA. In animals, TUTase-mediated uridylation of pre-miRNAs plays an important role in miRNA processing and the degradation of miRNA precursors (Heo et al., 2012; Kim et al., 2015). Recently, it has been shown that RNA tailing plays a role in the processing of pre-miRNAs in plants (Song et al., 2019). Extensive uridylation and cytidylation were detected at the 3’ ends of pre-miRNAs in Arabidopsis via 3’ RACE-sequencing. The uridylation of pre-miRNA is predominantly mediated by HESO1, whereas its cytidylation is mediated by HESO1 along with two additional NTPs, namely, NTP6 and NTP7 (**Figure 1**). Dysfunction of HESO1 led to a significant reduction in global median uridylation levels to below 1% in the *heso1* mutant, in contrast to approximately 8% in the Wt (Song et al., 2019). Pre-miRNA tailing was detected in both precisely and imprecisely processed termini, resulting in distinct outcomes. The highest tailing level was observed for the 1–3-nt truncated pre-miRNAs, in which the 3’ ends of the truncated pre-miRNAs were restored to their intact length (2-nt 3’ overhang), indicating a beneficial role of tailing in the processing of pre-miRNAs. For imprecisely processed pre-miRNAs with a 5’ overhang, HESO1 facilitates their degradation by appending long uridine tails (3-nt or longer). Moreover, a small portion of precisely processed intact pre-miRNAs undergo mono-uridylation/cytidylation, the functional implications of which remain unknown (Song et al., 2019). Although tailing restored truncated pre-miRNAs to their intact structure, the majority of them are non-productive in miRNA biogenesis, given the infrequent transfer of modifications from pre-miRNAs to mature miRNAs. Consistent with this, dysfunction of HESO1, the key NTP for pre-miRNA tailing, has minimal impact on the abundance of miRNAs (Zhao et al., 2012). However, a few miRNAs, such as miRNA158, miR163.1, and miR403, tailed pre-miRNAs could be processed into mature miRNAs, confirming the positive role of tailing in facilitating subsequent processing steps (Song et al., 2019).

The nascent miRNA/miRNA* duplex is the ultimate product after sequential slicing of pri-miRNAs by DCL1, which are methylated by HEN1 before being loaded onto AGO1 (Wang et al., 2019). The duplex structure of RNA is crucial for modulating the methyltransferase activity of HEN1 (Yu et al., 2005; Huang et al., 2009). In Phaseoleae species, the natural terminal mismatch in the miR1510/miR1510* duplex significantly hinders HEN1 activity on miR1510, while it does not affect the miR1510* strand (Fei et al., 2018). MiR1510 is mono-uridylated by HESO1, leading to the predominant accumulation of a 22-nucleotide isoform, which subsequently initiates the generation of phasiRNAs. Notably, the 22-nt miR1510 exhibited predominant methylation, suggesting the incidence of mono-uridylation on the miR1510/miR1510* duplex. Moreover, a recent study revealed that the miRNA/miRNA* duplex undergoes asymmetric modification by NTP4, a closely related homolog of HESO1 and URT1, which plays a role in regulating the abundance of miRNAs (**Figure 1**) (Kong et al., 2021). Dysfunction of NTP4 significantly reduced the overall abundance of miRNAs. Conversely, an increased abundance was detected for several corresponding miRNA*s, indicating contrasting functions of NTP4 in controlling the abundance of miRNA and miRNA* strands. *In vitro* investigations have shown that NTP4 can mono-uridylate the miR156* strand while not affecting the miR156 strand in the miR156/miR156* duplex. This finding suggested that NTP4 can perform asymmetric modification of the miRNA/miRNA* duplex. In addition, NTP4 preferred the 2-nt overhang at the 3’ end of the miRNA/miRNA* duplex. These data indicate that NTP4 might compete with HEN1 for the modification of nascent miRNA/miRNA* duplexes, thereby regulating the abundance of certain miRNAs. Consistent with this, NTP4-GFP shows exclusive nuclear localization where methylation of the miRNA/miRNA* duplex may also occur (Kong et al., 2021). *In vitro* studies have also demonstrated that HESO1 uridylates single-stranded and duplex sRNAs with varying levels of activity, thus highlighting the importance of the secondary structure of RNA substrates in influencing the activity of NTP (Kong et al., 2021).

#### 2.1.3 HESO1-mediated uridylation contributes to the stabilization of P4RNA

In plants, 24-nt het-siRNAs are the key regulators of RNA-directed DNA methylation (RdDM), a process that is essential for gene regulation and maintenance of genome stability (Matzke and Mosher, 2014). Several previous studies have identified non-templated nucleotides on the 3’ end of P4RNAs, which serve as the precursors of het-siRNAs (Zhai et al., 2015; Wang et al., 2016; Singh et al., 2019). A recent report revealed that HESO1 is responsible for the uridylation of P4RNAs (Ren et al., 2023). Dysfunction of HESO1 significantly impaired the uridylation of P4RNAs and led to a substantial reduction in P4RNA levels *in vivo*. This finding suggested that HESO1-mediated uridylation plays a crucial role in stabilizing P4RNAs and downstream siRNA generation (**Figure 1**). HESO1 is responsible for nearly all types of uridylation tails of P4RNAs. Disruption of HESO1 function leads to a reduction in mono-uridylation, a complete loss of di-uridylation, and a decrease in diverse U-containing tails, such as CU, UC, and AU (Ren et al., 2023). Furthermore, HESO1 prefers to tail P4RNAs terminated with adenine or uridine. Notably, in cases where the function of HESO1 is compromised, a variety of tail structures, although diminished, can still be detected at the 3’ end of P4RNAs, indicating that other NTPs and/or enzymes might also contribute to P4RNA tailing.

### 2.2 Uridylation plays an intricate role in mRNA metabolism

Uridylation of mRNAs is widespread and conserved among eukaryotes, with its primary role being to prime mRNA decay (Zuber et al., 2016; Morgan et al., 2017; Chang et al., 2018; Morgan et al., 2019). The degradation of mRNAs commences with deadenylation mediated by the Carbon Catabolite Repressor 4-Negative On TATA-less (CCR4-NOT) complex, which is followed by the removal of the 5’ m7G-cap structure through the decapping complex (Liu and Chen, 2016; Collart et al., 2023). Subsequently, mRNA is degraded by the 5’ -3’ exoribonuclease XRN4 and/or the 3’ -5’ SKI (SKI2-SKI3-SKI8)-exosome complex (Liu and Chen, 2016; Lange and Gagliardi, 2022). When the cytoplasmic mRNA degradation pathway is defective, the excessive accumulation of aberrant mRNAs may be sequentially processed by RDRs and DCLs, triggering a burst of spurious siRNAs which target endogenous mRNAs, consequently inducing posttranscriptional gene silencing (PTGS) (Branscheid et al., 2015; Martinez de Alba et al., 2015). Simultaneous disruption of bidirectional RNA decay pathways triggers extensive biogenesis of spurious siRNAs, resulting in dramatic endogenous gene silencing and consequential severe developmental defects (Zhang et al., 2015).

Uridylation has been detected in diverse types of mRNA molecules, such as full-length polyadenylated transcripts, deadenylated mRNAs, non-polyadenylated histone mRNAs, and 5’ mRNA fragments produced by RISC cleavage (Rissland and Norbury, 2009; Sement et al., 2013; Chang et al., 2014; Lim et al., 2014; Reimão-Pinto et al., 2016; Zuber et al., 2016). In plants, the process of mRNA uridylation has been studied mainly for the 5’ fragments and deadenylated mRNAs (Sement et al., 2013; Ren et al., 2014; Zuber et al., 2016, 2018; Scheer et al., 2021). Through the utilization of 3’ RACE-seq and TAIL-seq, a global view of mRNA uridylation and the relationship between uridylation and degradation of mRNAs have been elucidated in Arabidopsis (Zuber et al., 2016, 2018; Scheer et al., 2021). In addition to uridylation, TAIL-seq analysis revealed that approximately 5% and 2% of mRNAs in Arabidopsis were guanylated and cytidylated, respectively. Similar findings were described in humans, suggesting that the processes of guanylation and cytidylation of mRNA are evolutionarily conserved from plants to humans (Chang et al., 2014). However, the exact functions of these modifications have not been fully elucidated.

#### 2.2.1 Uridylation facilitates the degradation of 5’ fragments generated by RISC

Uridylation of 5’ fragments was initially identified in Arabidopsis and mice, and subsequently observed in human cells, indicating an evolutionarily conserved mechanism extending from plants to animals (Shen and Goodman, 2004; Xu et al., 2016). In Arabidopsis, HESO1 and URT1 catalyze the uridylation of 5’ fragments generated by RISC, which is composed of miRNAs and AGO proteins (**Figure 1**) (Ren et al., 2014; Zuber et al., 2018). The tailing characteristics exhibited by HESO1 and URT1 on the 5’ fragments closely resemble the uridylation pattern mentioned above for sRNAs. HESO1, the major TUTase responsible for uridylating 5’ fragments, recognizes substrates by interacting with AGO1. HESO1 can effectively synthesize short and long U-tails and promote the degradation of uridylated 5’ fragments. URT1 adds only one or two uridines to the 5’ fragments, which does not appear to function as a limiting factor in either the uridylation or destabilization of 5’ fragments (Zuber et al., 2018).

Several findings indicate that uridylated 5’ fragments can undergo degradation via the 5’–3’ RNA decay pathway. Uridylation appears to play a role in inducing decapping of the 5’ fragments and is correlated with the truncation of the 5’ products. This is evident because the proportion of uridylated 5’ fragments of *MYB33* is greater in the uncapped population than in the capped population in Arabidopsis (Shen and Goodman, 2004; Ren et al., 2014). The mechanism by which uridylation triggers decapping and subsequent 5’–3’ RNA degradation needs to be elucidated in future studies. Moreover, it has been demonstrated that uridylated 5’ fragments are subject to degradation via the 3’–5’ RNA decay pathway, facilitated by the activity of RISC-interacting exoribonucleases 1 and 2 (RICE1/2) (Zhang et al., 2017). RICE1 and RICE2 were identified as novel interaction partners of miRNA-RISC, interacting with AGO1 and AGO10. RICE1 and RICE2 eliminate the uridylated 5’ fragments resulting from RISC cleavage, thus playing a role in RISC dissociation and recycling (**Figure 1**).

#### 2.2.2 Uridylation favors 5’–3’ degradation of oligo(A)-tailed mRNAs and prevents excessive deadenylation of mRNAs

In eukaryotic cells, poly(A) tails play a pivotal role in regulating the stability of mRNAs. Deadenylation, the process of poly(A) tail shortening, serves as a rate-limiting step in the regulation of mRNA turnover (Eisen et al., 2020). Uridylation was predominantly observed on mRNAs with shortened poly(A) tails in mammals and plants (Sement et al., 2013; Chang et al., 2014; Zuber et al., 2016). In Arabidopsis, the median length of poly(A) tails in leaf tissues is 51-nt (Subtelny et al., 2014). In contrast, uridylated mRNAs, which represent approximately 32% of the total mRNAs, exhibit notably shorter poly(A) tails, usually containing fewer than 31 As, with the majority consisting of 10–25 As (Zuber et al., 2016; Scheer et al., 2021). URT1 is the main TUTase responsible for approximately 80% of mRNA uridylation. URT1 prefers to add 1–2 Us to deadenylated mRNAs with oligo(A) tails of approximately 13–15 As. This action serves to restore the tail length to 16-nt, a size that can be effectively recognized and bound by poly(A) binding protein (PABP) (**Figure 1**) (Sement et al., 2013; Zuber et al., 2016). Consistent with this, structural and enzymatic studies of URT1 have demonstrated that the residues L527 and Y592 confer a preference for purine interactions rather than pyrimidine interactions at the penultimate position of the RNA substrate. This preference regulates the specific addition of two uridine residues to the 3’ end of the oligo(A) tail (Zhu et al., 2020; Hu et al., 2022). It is hypothesized that URT1 is recruited by CCR4-NOT through interaction with ESSENTIAL FOR POTEXVIRUS ACCUMULATION 1 (EXA1) when poly(A) tails are insufficiently long to accommodate the final PABP, thereby promoting the uridylation of deadenylated mRNAs (Scheer et al., 2021). In *urt1*, 7% of the mRNAs remained uridylated. In addition, TAIL-seq analysis of *urt1* and *urt1 xrn4* validated the presence of redundant TUTase(s) responsible for mRNA uridylation (Zuber et al., 2016). URT1-independent uridylation is prone to occur on shorter oligo(A) tails containing fewer than 10 As and uridylation fails to restore the sufficient nucleotide extension length required for PABP binding. HESO1, the other prominent TUTase in Arabidopsis, has emerged as a good candidate for alternative uridylation of mRNAs. Further work is required to verify this potential.

URT1-dependent uridylation of mRNA plays a critical role in preventing excessive deadenylation. Recent research has indicated that two additive modes of action may restrict the accumulation of excessively deadenylated mRNAs. First, URT1 interacts directly with the decapping activator DECAPPING 5 (DCP5), establishing a molecular connection between the TUTase and decapping activators, consequently facilitating the 5’–3’ elimination of deadenylated mRNAs with oligo(A) tails of 10–25 As (Scheer et al., 2021). Consistent with this observation, there is an accumulation of uridylated mRNAs in the *xrn4* mutant (Sement et al., 2013; Zuber et al., 2016). Furthermore, the *heso1 urt1 ski2* triple mutant exhibits developmental defects, indicating the involvement of mRNA uridylation in the 5’–3’ degradation process (Wang et al., 2022). Second, uridylation intrinsically prevents excessive deadenylation by impeding the deadenylase activity of CAF1, a core subunit of the CCR4-NOT complex (Scheer et al., 2021). Impeding deadenylation at the 3’ end may contribute to establishing the 5’–3’ directionality of mRNA degradation, a process crucial for co-translational mRNA decay. A previous study consistently demonstrated that the protection conferred by URT1 at the 3’ end can operate on polysomes (Sement et al., 2013). In addition, the binding of PABP to uridylated mRNAs could serve as a protective mechanism against 3’ ribonucleolytic degradation of mRNAs. This interaction may also play a role in determining the 5’–3’ directionality of mRNA degradation and potentially contribute to mRNA storage (Zuber et al., 2016). Moreover, mRNA uridylation that is independent of URT1 also serves a unique function in mRNA metabolism. URT1-independent uridylation may be involved in the 5’–3’ decay of deadenylated mRNAs, since uridylation is elevated in *urt1 xrn4* compared to *urt1* (Zuber et al., 2016). Moreover, considering the redundant functions of HESO1 and URT1 in mRNA degradation (Wang et al., 2022), the likelihood that HESO1 mediates URT1-independent uridylation is further enhanced. Overall, the uridylation of mRNAs plays a multifaceted role in mRNA metabolism.

#### 2.2.3 Uridylation-dependent mRNA decay plays an important role in RNA surveillance

The degradation of mRNAs plays a fundamental role in RNA quality control (RQC), which serves as a surveillance mechanism to selectively discriminate and eliminate various aberrant RNAs, including truncated, non-polyadenylated, and uncapped mRNAs (Liu and Chen, 2016). Several recent studies have suggested that mRNA surveillance is linked to uridylation-dependent mRNA decay. Disruption of uridylation-dependent RNA decay elicits the synthesis of spurious siRNAs, which seriously impacts plant development (Scheer et al., 2021; Wang et al., 2022). Simultaneous dysfunction of HESO1, URT1, and SKI2 induced the biogenesis of illegitimate siRNAs from the Calvin cycle gene *TRANSKETOLASE 1* (*TKL1*) locus, resulting in PTGS of *TKL1* and leaf etiolation because of diminished photosynthetic efficacy (Wang et al., 2022). Mutation of *RDR6* largely rescued the molecular and physiological defects in *heso1 urt1 ski2*, further demonstrating the involvement of the PTGS pathway following the impairment of uridylation-dependent mRNA decay. In addition, URT1 uridylates a subset of deadenylated mRNAs and guides them toward the 5’–3’ decay pathway by directly interacting with DCP5. Simultaneous dysfunction of URT1 and XRN4 resulted in siRNA-mediated PTGS of endogenous mRNAs and adversely affected development, thus underscoring the importance of URT1-mediated uridylation in mRNA surveillance (Scheer et al., 2021). Consistent with the aforementioned studies, URT1 was recognized as a suppressor of siRNA-mediated PTGS of transgenes and specific endogenous transcripts (Li et al., 2019).

The incorporation of RNA uridylation into the RNA surveillance pathway suggests substantial potential for uridylation playing a role in facilitating RNA decay. Notably, HESO1 and URT1 function redundantly or cooperatively in the degradation of *TKL1* mRNA, as evidenced by the absence of a leaf etiolation phenotype in *heso1 ski2* and *urt1 ski2* mutants, unlike in *heso1 urt1 ski2* (Wang et al., 2022). As previously mentioned, URT1 mainly promotes the uridylation of deadenylated mRNA, whereas HESO1 is the primary enzyme that uridylates sRNAs and 5’ fragments. Two hypotheses have been proposed regarding the relationships between HESO1 and URT1. First, HESO1 and URT1 redundantly uridylate *TKL1* mRNA. Simultaneous impairment of HESO1 and URT1 results in the complete abolition of *TKL1* mRNA uridylation and triggers siRNA biogenesis in *ski2*. Second, HESO1 aids in the clearance of *TKL1* mRNA fragments that are cleaved by primary siRNAs generated in *urt1 ski2*. Dysfunction of HESO1 in the *urt1 ski2* background leads to heightened accumulation of cleaved *TKL1* mRNA fragments, which potentially serve as templates for the generation of secondary siRNAs. Importantly, these possibilities are not mutually exclusive. The uridylation mediated by HESO1 and URT1 may play redundant and distinct roles in the control of mRNA stability (Wang et al., 2022).

### 2.3 Prevalent uridylation of phytoviral RNAs with various patterns

Uridylation facilitated by host enzymes is a prevalent phenomenon when eukaryotic RNA viruses infect both lower eukaryotes (fungi) and higher eukaryotes (plants and animals) (Huo et al., 2016). *Caenorhabditis elegans* CDE-1 and human TUT4/7 mark the viral RNA genome of Orsay virus and mRNAs of influenza A virus, respectively, for degradation, which indicates the significant involvement of RNA uridylation in antiviral mechanisms in animals (Le Pen et al., 2018). Recently, a large-scale view of phytoviral RNA uridylation was profiled for representatives of the main families of positive single-stranded RNA phytoviruses by 3’ RACE-seq (Joly et al., 2023). Uridylation was detected in all 47 viral RNAs investigated, with levels ranging from 0.2%–90%, revealing the prevalence and diversity of uridylation patterns among phytoviruses. Polyadenylated phytoviral RNAs of grapevine fanleaf virus (GFLV) exhibit strict mono-uridylation, which is independent of HESO1 and URT1, confers a strategic advantage to the virus. Structured RNAs, particularly those with occluded 3’ ends, have the potential to hinder the uridylation of specific physiological substrates by TUTases (Warkocki et al., 2018). Consistent with this, phytoviral RNAs with tRNA-like structures (TLSs) exhibit low levels of uridylation, with proportions ranging from 0.3%–2.2%. The host TUTases HESO1 and URT1 have been shown to share certain viral RNA substrates, yet they exhibit differential uridylation patterns on these viral RNAs. Similar to the uridylation pattern observed in the mRNAs and sRNAs of Arabidopsis, HESO1 prefers to add long U-tails, while URT1 shows a bias toward substrates that are terminated with adenine (Zhu et al., 2020). In addition, HESO1 predominantly targets turnip mosaic virus (TuMV) RNAs with shorter oligo(A) tails, typically with a median length of 4-nt, while URT1 preferentially targets TuMV RNAs with oligo(A) tails that are approximately 10–11-nt in length. The observed tailing pattern resembles the characteristics identified in mRNA uridylation, thereby reinforcing the likelihood of HESO1 functioning as a TUTase uridylating mRNA with shorter oligo(A) tails (Zuber et al., 2016). This finding also suggested a role for HESO1 and URT1 in directing viral RNAs toward degradation. Notably, uridylation frequently occurs on turnip crinkle virus (TCV) and TuMV degradation intermediates, and the accumulation of TCV RNA degradation intermediates increased in the *heso1 urt1* double mutant, indicating a positive role of uridylation in the clearance of viral RNA. Furthermore, in addition to uridines, tails consisting of other nucleotides were also observed in viral RNAs, suggesting a consistent role of NTPs in the process of tailing of both exogenous and endogenous RNAs. Collectively, the widespread uridylation observed in phytoviral RNAs demonstrates notable variability and adheres to virus-specific patterns, revealing an unforeseen intricacy in uridylation within phytoviral RNA metabolism.

## 3 Functions of NTPs in plant development and stress responses

The molecular functions of several plant NTPs have been elucidated through a combination of genetic and biochemical approaches. However, their roles in plant growth and development have received less extensive research attention, primarily focusing on the functions of HESO1 and URT1. The rice homolog of *HESO1* was demonstrated to modulate heading date through a genome-wide association study (GWAS) (Yano et al., 2016; Tanaka et al., 2021). The single nucleotide polymorphism (SNP) in the HESO1 homolog results in an amino acid substitution between haplotypes A and B. Varieties harboring haplotype A (valine) in the HESO1 gene, which is identical to that found in Arabidopsis, exhibited earlier heading dates than those harboring haplotype B (isoleucine). In Arabidopsis, HESO1 and URT1 regulate the growth and development of seedlings by collaborating with cytoplasmic RNA degradation factors for RNA surveillance. The *urt1 xrn4* double mutant exhibited a failure to initiate leaf formation and was unable to progress to the development of inflorescences (Scheer et al., 2021). The *heso1 urt1 ski2* triple mutant displayed symptoms of leaf etiolation (Wang et al., 2022). Arabidopsis *ntp* mutants including *heso1* and *urt1* do not display discernible phenotypic traits when grown under standard conditions (Yang et al., 2017). Given the importance of NTPs in RNA metabolism and their conservation in the plant kingdom, studies have been carried out to analyze the expression patterns of *NTPs* under different stress conditions in rice, maize, and soybean (Yang et al., 2017; Lu et al., 2018; Kang et al., 2024). In these crops, various NTPs exhibit expression patterns that are specific to certain tissues or developmental stages. The analysis of gene expression and RNA-seq data revealed notable variations in the levels of *NTP* expression under different abiotic stresses, such as high salt, drought, and cold stress. This information serves as a fundamental basis for further functional investigations. In addition, an extensive survey of phytoviral RNAs revealed the widespread presence of 3’ tailing (Joly et al., 2023). In animals, mono-uridylation mediated by TUT4/7 promotes the degradation of viral RNA, whereas mixed tailing mediated by TENT4A/B contributes to the stabilization of viral RNA (Le Pen et al., 2018; Yeo and Kim, 2018; Kim et al., 2020). The functions of NTPs in host–virus interactions merit detailed examination, given that phytoviral RNAs can be adenylated, cytidylated, guanylated, uridylated, or tailed with a combination of nucleotides.

## 4 Future perspectives

In the last decade, non-canonical tailing mediated by NTPs has been recognized as a crucial step in the posttranscriptional modulation of gene expression. The principal role of RNA uridylation is to regulate RNA stability. Mono-/di-uridylation and oligo-uridylation (3-nt or more) seem to direct the corresponding substrates toward distinct metabolic pathways, as evidenced by the identification of various RNA species. The addition of mono-/di-U tends to restore the sequences of RNA substrates and facilitate subsequent processing. However, oligo(U) typically functions as a degradation signal that initiates the decay of substrate RNAs. To date, the function of non-U tails in RNA metabolism remains largely unclear. As new RNA substrates are being discovered, growing evidence suggests that several NTPs involved in the tailing process remain uncharacterized. Adequate genetic backgrounds are required to present a comprehensive perspective on non-canonical tails. Despite HESO1 and URT1 being well characterized, there are still numerous unresolved questions that require further elucidation. In accordance with their respective functions, HESO1 is localized in both the cytoplasm and nucleus, whereas URT1 exhibits cytosolic localization (Ren et al., 2012; Wang et al., 2015). HESO1 and URT1 exhibit unique substrate preferences and distinctive tailing characteristics. The crystal structure of URT1 has been elucidated, revealing its molecular mechanism in tailing (Zhu et al., 2020; Hu et al., 2022). The molecular functions of HESO1 can be more comprehensively understood by elucidation of its protein structure. HESO1 uridylates viral RNAs that have shorter oligo(A) tails. It is of interest to investigate whether HESO1 is also involved in the uridylation of endogenous mRNAs with shorter tails (<10 As) in Arabidopsis. Moreover, the mechanisms by which URT1 and HESO1 are recruited to oligo(A) tails of varying sizes and the associated cofactors remain unclear. The frequency of uridylation shows a negative correlation with the overall mRNA half-life (Chang et al., 2014; Scheer et al., 2021). The uridylation of mRNAs mediated by URT1 establishes the 5’–3’ polarity of degradation. The URT1-independent long U-terminal region of mRNA could be considered a signal for degradation. What are the “readers” that recognize U-tails of mRNAs in plants? The LSm1-7 complex exhibits a preference for binding short oligo(A) tails (<10 As), and it is proposed that uridylation promotes the decapping of deadenylated mRNAs in mammalian cells by facilitating the binding of the LSm1-7 complex (Chowdhury et al., 2007; Montemayor et al., 2020). Uridylated short oligo(A) tails that are independent of URT1 are likely targets of the LSm1-7 complex. This assumption is based on the potential that URT1 could directly interact with decapping activators, thus circumventing the necessity for LSm1-7 binding. Alternatively, the 3’–5’ exonuclease DIS3L2 can directly recognize and preferentially target oligo(U) tails of mRNAs in animals (Lubas et al., 2013; Malecki et al., 2013; Wu et al., 2023). However, whether SUPPRESSOR OF VARICOS (SOV), the plant homolog of DIS3L2, functions on uridylated mRNAs remains to be established.

RNA tailing is generally believed to have developed as a mechanism to facilitate the degradation or turnover of unnecessary transcripts (Yu and Kim, 2020). Our understanding of the physiological functions of NTPs, which are key players in shaping both the coding and noncoding transcriptomes, in plant growth, development, stress responses, and disease resistance is still very limited. Programmed uridylation-primed mRNA decay plays a crucial role in reproductive processes in animals (Chang et al., 2018; Morgan et al., 2019). The uridylation profiles of RNAs and their functions in specific plant developmental stages, particularly in plant reproductive stages are worth investigating. Furthermore, RNA tailing mediated by NTPs can effectively regulate the transcriptome in a timely manner in response to various stresses. The identification and expression analysis of NTPs under various abiotic stress conditions have been carried out in several crops. Further investigations will aid in elucidating the roles of NTPs in plant stress responses.
